# Understanding the challenges to implementing case management for people with dementia in primary care in England: a qualitative study using Normalization Process Theory

**DOI:** 10.1186/s12913-014-0549-6

**Published:** 2014-11-08

**Authors:** Claire Bamford, Marie Poole, Katie Brittain, Carolyn Chew-Graham, Chris Fox, Steve Iliffe, Jill Manthorpe, Louise Robinson, the CAREDEM team

**Affiliations:** Institute of Health and Society, Newcastle University, Baddiley Clark Building, Richardson Road, Newcastle, NE2 4AX UK; Research Institute, Primary Care and Health Sciences, Keele University, Keele, Staffordshire ST5 5BG UK; Norwich Medical School, University of East Anglia, Norwich Research Park, Norwich, NR4 7TJ UK; Department of Primary Care and Population Health, University College London, Royal Free Campus, Rowland Hill St, London, NW3 2PF UK; Social Care Workforce Research Unit, King’s College London, Strand, London, WC2R 2LS UK

**Keywords:** Dementia, Case management, Patients, Carers, Primary care, Normalization Process Theory

## Abstract

**Background:**

Case management has been suggested as a way of improving the quality and cost-effectiveness of support for people with dementia. In this study we adapted and implemented a successful United States’ model of case management in primary care in England. The results are reported elsewhere, but a key finding was that little case management took place. This paper reports the findings of the process evaluation which used Normalization Process Theory to understand the barriers to implementation.

**Methods:**

Ethnographic methods were used to explore the views and experiences of case management. Interviews with 49 stakeholders (patients, carers, case managers, health and social care professionals) were supplemented with observation of case managers during meetings and initial assessments with patients. Transcripts and field notes were analysed initially using the constant comparative approach and emerging themes were then mapped onto the framework of Normalization Process Theory.

**Results:**

The primary focus during implementation was on the case managers as isolated individuals, with little attention being paid to the social or organizational context within which they worked. Barriers relating to each of the four main constructs of Normalization Process Theory were identified, with a lack of clarity over the scope and boundaries of the intervention (*coherence*); variable investment in the intervention (*cognitive participation*); a lack of resources, skills and training to deliver case management (*collective action*); and limited reflection and feedback on the case manager role (*reflexive monitoring*).

**Conclusions:**

Despite the intuitive appeal of case management to all stakeholders, there were multiple barriers to implementation in primary care in England including: difficulties in embedding case managers within existing well-established community networks; the challenges of protecting time for case management; and case managers’ inability to identify, and act on, emerging patient and carer needs (an essential, but previously unrecognised, training need). In the light of these barriers it is unclear whether primary care is the most appropriate setting for case management in England. The process evaluation highlights key aspects of implementation and training to be addressed in future studies of case management for dementia.

## Background

The 2011 World Alzheimer Report highlighted the need for early intervention in dementia and suggested collaborative care as a possible means of improving the quality and cost-effectiveness of community care [1]. Collaborative care comprises a care/case manager who co-ordinates care between professionals, liaises between primary and secondary care, and utilises evidence-based care pathways to address physical and psychosocial needs. It has been found to be effective in conditions such as depression [2,3]; however the evidence for dementia is mixed. While earlier trials found significant benefits for people with dementia and family carers (caregivers) [4,5]; recent systematic reviews found little clinical or cost-effectiveness evidence to support widespread case management implementation [6], beyond some quality of life benefits [7,8].

Around two-thirds of people with dementia in England live at home, supported by their family, health and social care practitioners and specialist dementia services, such as Admiral Nurses^a^ [9]. Concerns over the quality [10] and rising costs of dementia care have led to calls for more integrated, cost-effective approaches [11]. In the UK care management is provided through social care and community mental health services, but is usually time-limited and reactive. We therefore adapted a successful case management intervention [12], from the United States (US) for primary care in England [13] and evaluated the acceptability of this model for people with dementia and their carers (CAREDEM study). The results are reported elsewhere [14], but a key finding was that very little case management took place.

Relatively few studies of case management have included a process evaluation or explored the characteristics of case management and the factors which influenced implementation [15-17]. The UK Medical Research Council guidance for developing and evaluating complex interventions recommends conducting a process evaluation to provide insight into unexpected outcomes, intervention fidelity and factors influencing implementation [18]. The primary purpose of the process evaluation conducted as part of the CAREDEM study was to explore the perceived value and benefits of case management and the feasibility and acceptability of case management in practice [19]. When low levels of implementation became apparent, we extended our work to consider fidelity, reach and dose of case management [19]. We used the theoretical framework of Normalization Process Theory (NPT, [20-22]) to explore the social processes and organizational issues that influenced the practical implementation of case management. NPT considers the practical work involved in making sense of the intervention (*coherence*), investing in the intervention (*cognitive participation*), delivering the intervention (*collective action*) and modifying and embedding the intervention to suit local circumstances (*reflexive monitoring*) [20]. Using this theoretical framework we illuminate the reasons why a dementia case management intervention, successful in a US study, proved difficult to deliver within primary care in England.

## Methods

The CAREDEM study was designed to test the transportability of a US case management model to primary care in England [12]. The model and associated materials were adapted for use in England by a multi-disciplinary group, including family carers and representatives of voluntary organisations [13]. The materials included a job description and a list of desirable and essential attributes for a case manager; an educational needs assessment to inform training and mentoring; and written information designed to be used with carers and people with dementia (the ‘manual’) [13]. An overview of the tasks to be undertaken by the case managers is provided in Table 1.

**Table 1:** **Tasks to be undertaken by case managers**

1	Identify people with dementia (PWD) from general practice lists
2	Review medical records of PWD +/− their carer(s), noting any gaps in the record and also the involvement of other possible sources of support
3	Liaise with other professionals who know the PWD to learn their perspectives on individual or family needs
4	Engage with the PWD +/− carer to identify their main concerns or unmet needs
5	Update or fill in gaps in GP medical records and where appropriate update social care records
6	Analyse information obtained with PWD & carers
7	Map support available to and wanted by PWD & carer. Create a personal care or support plan with each PWD & carer, and initiate actions that will provide that support
8	Analyse information obtained with other relevant practitioners
9	Prioritise individual PWD and carers: Assess need for action in terms of ‘intensive’, ‘maintenance’ and ‘holding’
10	Build the care plan into the GP medical records, and share with other professionals and agencies as needed
11	Organise systematic follow-up to review the outcomes of actions taken, meet regularly with the GP or other relevant clinical leads, and act as an advocate for the PWD and carers
12	Meet regularly with his/her mentor, to discuss PWD and carers with whom they are working, to review prioritisation, to resolve any problems that have arisen and to plan the end of their role with the PWD and their carers, as appropriate
13	Undertake professional updating and top-up training, as needed
14	Meet with and communicate with members of the research team to discuss the case manager role as it develops

Four case managers were recruited (two practice nurses and two social workers). Only three case managers were involved in face-to-face contact with patients and carers (one left post before the intervention began). Each case manager had an induction session (with the case manager mentor (‘mentor’ hereafter) and/or the principal investigator) which focused on building relationships and identifying competencies and training needs. On-going training and support was provided by the mentor (an experienced Admiral Nurse). People with dementia were identified from primary care Quality and Outcomes Framework (QOF) dementia registers and electronic searches. Their eligibility was assessed by case managers in conjunction with practice staff. Inclusion criteria were (1) having a dementia diagnosis confirmed by specialist services; (2) having a carer; (3) not being resident in a care home; and (4) not having regular reviews by specialist services.

The embedded process evaluation used ethnographic methods - in-depth qualitative interviews, informal discussions and observations - to explore factors influencing the delivery of case management in practice. Interviews were conducted with a range of stakeholders (Table 2) to explore different perspectives on case management. In total, 49 stakeholders were interviewed; the majority face-to-face, with a small number by telephone. At least one GP and a practice manager was interviewed from all but one participating practice. Health and social care professionals outside the practice were identified through observation and informal discussions with case managers. While some of the health and social care professionals interviewed had direct contact with the case managers, others gave a more theoretical perspective on case management (e.g. a commissioner/funder). In recruiting patients and carers we aimed to include people with a range of scores on the baseline measures (including activities of daily living; quality of life; and cognitive function) and to ensure a range in terms of age, gender and relationship between the patient and carer. Where possible separate interviews were conducted with patients and carers to facilitate exploration of different experiences, however some participants requested a joint interview.

**Table 2:** **Stakeholder interviews (n =49)**

**Stakeholder**	**Number of interviews**
Person with dementia	6
Carer	10
Case manager	9
Case manager mentor	4
Research team members	2
General practitioner	6
Administrative practice staff	5
Community mental health team	2
Voluntary sector workers	3
Commissioners/funders	2

Interviews were conducted throughout the feasibility study to capture key events, such as the induction session with case managers, and to understand the processes and experiences of case management at different times. Case managers and their mentor were therefore interviewed on several occasions. Separate topic guides were developed for each stakeholder group and were adapted throughout data collection in light of the levels of intervention delivered and emerging themes. Key areas covered in the interviews were:

- Induction content and process
- Understanding of the case manager role
- Expectations of case management
- Supervision (frequency, content)
- Outcomes of case management
- Views on process of case management (timing, intensity, type of contact)
- Views on case managers (professional background, skills)
- Advantages/disadvantages of primary care base

We observed case manager induction; one new patient/carer assessment undertaken by each case manager; telephone calls between case managers and patients/carers; and wrote reflective field notes on baseline and follow-up patient/carer assessments (scheduled prior to commencement of case management and approximately five months later). Due to the geographical dispersion of the case managers, they met only twice as a group during the study; both meetings were observed, apart from a short, closed session between the mentor and case managers at each meeting. Reflective field notes were also written about team meetings, informal discussions and telephone conversations with case managers and primary care team members, visits to general practices and interviews.

Ethics committee approval for this study was obtained (NRES Wandsworth 11/LO/1555) and written informed consent was obtained from all participants.

### Data management and analysis

All interviews were audio-recorded, transcribed, checked and anonymised. Analysis began with individual team members (CB, KB, MP, LR) reading and re-reading a number of transcripts to familiarise themselves with the data and identify preliminary themes. We then held a series of data workshops in which we discussed our emerging ideas and developed a draft coding frame. We applied this to a small number of transcripts and the findings were discussed in subsequent data workshops. Following a series of iterations, a final coding frame was agreed. All transcripts were then coded in Nvivo to facilitate data management.

We then reviewed the coded data for different stakeholder groups; this led to the combination of some codes, the identification of new sub-codes and the production of a narrative summarising the key themes for each group. These narratives were compared and overarching themes identified across the stakeholder groups. The resulting themes were mapped to the four main constructs of NPT. Rather than comprehensively coding field notes and other documents (e.g. notes of case manager meetings), we read through these documents noting examples of the codes, creating new codes and modifying existing codes as needed [23].

Illustrative data included in the results are identified by type of participant (patient, carer, case manager, mentor, research team member or health care professionals). Quotations from patients, carers and professionals are also identified by site (A, B or C). Although we recruited male and female case managers, to maintain confidentiality we have referred to all case managers as female and have not identified them by site. By virtue of her role, the mentor was identifiable and she has reviewed and agreed to the use of all data attributed to her.

## Results

The limited implementation of case management reflected: a lack of clarity over the scope and boundaries of the intervention; variable investment in the intervention; a lack of resources, skills and training to deliver case management; and limited reflection and feedback on the case manager role. These key themes map onto the four main constructs of NPT (Table 3, [20]).

**Table 3:** **Mapping of overarching themes and subthemes to NPT framework**

**NPT construct**	**Theme**	**Subthemes**
Coherence	Making sense of the case manager intervention	Perceived value of the concept of case management
		Clarity over the case manager role
Cognitive participation	Investment in case management	Practice investment in case management
		Investment by case managers
		Fit of case management with existing skill-sets
Collective action	Implementing case management in practice	Time available for case management
		Implementation in research vs clinical practice
		Support and supervision of case managers
Reflexive monitoring	Appraising and embedding of case management	Assessing the impacts of case management
		The ‘right’ intervention but at the wrong time
		Embedding case management in practice

### Coherence: making sense of the case manager intervention

An understanding of the scope and nature of an intervention is fundamental to its delivery in real world practice and its subsequent evaluation. While the concept of case management had an intuitive appeal to most participants, many revealed uncertainties about the scope and aims of the intervention and its overlap with existing roles.

#### Perceived value of the concept of case management

Nearly all stakeholders viewed the concept of case management as worthwhile. People with dementia and carers saw case managers as the first point of contact for information who could facilitate access to other services thus avoiding the need and effort to engage with multiple agencies:

*“It’s the most appropriate place that she’s there attached to the GP [general practitioner] and let’s face it I can’t get any service for mum or any care unless I go through that point.”* (Carer, A06)

All stakeholders valued the continuity of care potentially offered by case management, especially since this was rarely provided by other services. Professionals viewed the opportunities for early and on-going support as potentially improving outcomes both for patients and carers:

*“We do know that people with dementia, their needs change dramatically over time in different ways and ideally we would keep them on and monitor and follow-up and provide support as their needs do change, but that’s impossible. So they have to be discharged, and then hopefully are re-referred before a crisis occurs but sadly it’s often at the point of crisis so that’s one area where the case manager in primary care can plug that gap.”* (Old age psychiatrist, B07)

Basing case management in primary care was seen as appropriate by most stakeholders since attending the GP surgery was seen as an ‘ordinary’ activity compared to attendance at hospital or contacting local authority social services. Participating practices identified a number of potential benefits of case management, for example, saving appointment time with the GP and having access to a ‘dementia specialist’ in the practice:

*“I think several things for the patients and their carers, someone in the practice who was known to them as having an interest in dementia and would be a point of contact for them.”* (GP, B04)

#### Clarity over the case manager role

Despite enthusiasm for the concept of case management, all stakeholders expressed a lack of clarity over the remit of case managers. People with dementia and carers were uncertain about the specific areas of support available:

*Int: “Do you think a case manager could have helped to support you in that role,*

*Carer: Eh, I don’t know, is it their job to do that, is it?”* (Carer, C53)

Case managers themselves expressed uncertainty over the boundaries of their role, particularly at the beginning of the project:

*“Certainly in the early stages it didn’t seem very clear at all to me what was expected or what I was supposed to be doing, or how I was supposed to go about it…. I didn’t really fully understand what I was doing.”* (Case manager 1)

Although in theory professionals could see that case management potentially ‘plugged a gap’ in services, concerns were expressed over potential duplication with existing roles and services:

*“I think that the problem sometimes is that roles aren’t clearly, or maybe not clearly defined enough, between, for example, our community mental health nurses, the dementia advisor that we work with, and the case manager.”* (Old age psychiatrist, B07)

Even within the research team there was no single, consistent view on the intervention; whereas one team member described the role as being to “implement the manual”; others saw it as a “diluted” version of the Dementia UK Admiral Nursing service. This uncertainty was reflected both in field notes and interviews:

*“I was left feeling very unclear what exactly is going to be delivered by the case managers and how this relates to the protocol (and the manual).”* (Field notes, 11.7.2012, meeting with mentor & case manager 3)

*“I think there’s a big question about what we mean by case management, really I don’t think there’s any real clarity about what the term means.”* (Interview with research team member)

### Cognitive participation: investment in case management

At the outset of the feasibility study, espoused commitment to the intervention was high amongst case managers, participating practices and research team members. However, this initial enthusiasm did not always translate into sustained investment.

#### Practice investment in case management

There was little leadership or involvement of members of the primary care team in promoting or engaging with the intervention. This was particularly true of practices where the role of case manager was taken on by a practice nurse^b^. Where the case manager was seconded from local authority social services, the practices held an initial meeting at which the case manager was introduced and described her role. However, there was little contact between the primary care team and the case manager beyond this initial briefing meeting:

*“[Case manager] mentioned that she was not having much success in her attempts to engage with [name of practice]. [Case manager] had met with the lead GP and found her very enthusiastic, but mentioned that recently neither the lead GP nor the practice manager were currently returning her calls.”* (17/7/2012, observation of induction session with case manager 3)

The ‘fit’ of case management with the culture and time scales typical of primary care raised questions over whether this was the right setting for the intervention:

*“The surgery is set up for everybody to have 10 – 15 minutes appointments with however many people you can cram into your day, it’s not about sitting and reflecting and analysing and spending a lot of time on a person and within that person a particular problem for that particular person […] it’s not how a GP surgery functions.”* (Case manager 4)

Recruitment of patients and carers proved difficult in all practices. While GPs in one practice suggested potential participants, the rigid interpretation of inclusion criteria by the case manager meant that only patients who were already on the practice dementia register were eligible. The failure to capitalise on the interest shown by the GPs in referring patients may have affected commitment to the study within this practice:

*“I mean they showed some initial interest in referring cases and I think if they could have done that it would have been very different but obviously they couldn’t, we weren’t open to the GPs referring people in so I think […] that they really didn’t bond with it.”* (Case manager 3)

#### Investment by case managers

Some case managers saw the role as an opportunity to develop their skills and expertise in dementia. However, others did not have the same enthusiasm or commitment:

*“I was nominated and I had the right to refuse but I was nominated […] I suppose as soon as they got a whiff of my case management experience my fate was sealed.”* (Case manager 4)

The ability of case managers to respond to challenges arising during the study also indicated their investment in the role. For example, time constraints were a recurrent problem for the practice nurses. While one practice nurse seemed overwhelmed by constant interruptions, the other was more pro-active and, by repeatedly reminding her colleagues that she was not available on one afternoon each week, this case manager successfully protected her case management afternoon to the extent where she planned to continue to use this time for dementia-related activities after the end of the study:

*“[Case manager] is keen to develop her role more and it has already been agreed with the practice that she will take over responsibility for the practice dementia register and patient reviews. She is going to run dementia review clinics once a month and will also do some home visits to review people who are housebound or would find it difficult to get to the surgery.”* (10/12/2012, field notes of informal discussion with case manager 1)

Other case managers seemed less able to constructively address the challenges encountered, sometimes attributing problems to causes outside their control. For example, one case manager attributed low levels of the intervention to the nature of the patients recruited to the study:

*“I suspect we will be getting people who actually are fairly well off and managing quite well and have the time and inclination to help out. The people who are really struggling, who are in complete crisis, chaos, who could do with the service, ironically they’re going to be the ones who are going to be like ‘Ah for God’s sake, just no. I just can’t be doing with that on top of everything else’.”* (Case manager 3)

#### Fit of case management with existing skill-sets

The case managers brought different skills, professional backgrounds and experiences to their role. While all stakeholders viewed knowledge of dementia as crucial, two of the case managers had more generic skills and the feasibility of developing sufficient expertise within the time available was questioned by the mentor:

*“For me one of the fundamental flaws was perhaps recruiting people into a dementia case management role where not everyone had a basic understanding around dementia.”* (Mentor)

Knowledge of local services had also been seen as central to the case manager role; while the case managers with a social work background already had a good knowledge of local services, developing this knowledge was challenging for practice nurses:

*“I’ve learnt a lot about different services and networking and I feel like there’s a lot to learn still, loads and loads and loads, I still feel 'oh, I don’t know who to turn to with this'.”* (Case manager 1)

Patients and carers emphasised the good interpersonal skills of case managers, commenting on their ‘empathy’; ‘ability to listen’; ‘making people feel at ease’ and ‘not rushing people’. Such skills were seen as key to developing relationships:

*“Going into someone’s home and assessing the situation fully takes a lot of time and my own experience is that carers are often a little bit anxious about what you’re going to suggest and what you’re going to try and do and whether you’re going to be critical of - they might know that their care is falling short of the ideal a lot of the time but it’s their husband or their wife and they want to carry on. I think people are very scared and it takes a while to build up a relationship, to actually develop that.”* (GP, B04)

### Collective action: Implementing case management in practice

The main barrier to the implementation of case management was the mismatch between the skills and resources available and those required to deliver the intervention. The situation seemed to be exacerbated by participation in a feasibility study.

#### Time available for case management

The time allocated to case management differed significantly across research sites. While one full time social worker covered two GP practices; the practice nurses in the other sites were allocated only half a day a week for case management. While the practices were reimbursed for the practice nurses’ time, there was little evidence that this money had been used to provide cover for their usual nursing duties. Consequently their primary care colleagues rarely recognized the legitimacy of time spent on case management, creating additional difficulties:

*“with (Case Manager 4) for example, the last time I went to see her she was pulled out to do two practical procedures in the middle of our meeting, even though they knew I was doing supervision”* (Mentor)

Despite these concerns, carers did not necessarily perceive the limited time available to the case manager as a barrier to seeking help if needed:

*“I think [case manager] is a very busy woman so to pile things onto her would be wrong but it’s lovely knowing she’s there if I need her, I can pick that phone up, I can even ring her at the surgery and she’d listen which is nice.”* (Carer, B03)

The time allocated for case management (both in terms of the hours per week and the duration of the feasibility study) inevitably impacted on the development of therapeutic relationships with patients and carers. The majority of patients and carers had only a single contact with their case manager; where there were a number of contacts, most were by telephone. While this was acceptable to most participants, some felt face to face contact was needed to establish a relationship:

*“So that then when it gets to a stage when we really do need help, we've got the confidence in the person you’ve been seeing all along.”* (B04, carer)

While home visits were time intensive, particularly in the rural area, they were thought to provide a better understanding of participants’ social environment:

*“You see the nicest turned out people who they make an effort, they come into the doctors but you go home and then you realise there’s mouldy food in the fridge… you walk in and you get a picture straight away usually, particularly about circumstances.”* (GP, C02)

The time available did not entirely explain the limited amount of case management that took place. Despite having five times more sessions allocated to each practice, the case manager from a social work background had fewer contacts with patients and carers than one of the practice nurses, and had minimal contact with the GPs in participating practices:

*“If I’m honest we hardly ever saw [case manager] within the practice. We only saw her twice, once at the very start and once after I think I’d met with you and said we haven’t heard or seen her and she then turned up once again.”* (GP, C52)

#### Implementation in research versus clinical practice

Participating in a research project created additional work for case managers since they were responsible for maintaining a recruitment log and (for practice nurses) making the initial contact with eligible patients and carers. The familiarity of the research team with recruitment processes and operationalizing inclusion and exclusion criteria meant that we underestimated the challenges of these research tasks for the ‘research naïve’ case managers. While there was some recognition amongst the research team of the unrealistic expectations on case managers, there were some tensions between the case managers and the research team over both the workload and underlying values:

*“I felt that sometimes I was getting lots of emails from people saying ‘could I do this, could I do that, have you done the log, have you done this paperwork?’ […] I didn’t think people really understood the role of a practice nurse.”* (Case manager, 1)

*“I think it’s been quite hard to avoid the fact that ultimately this has been for the benefit of the study not for the service users or patients and carers etc. And I think […] there has been a feeling that the participants don’t really matter and that they are just tools to gather this information to get a research paper out of it.”* (Case manager, 3)

#### Support and supervision of case managers

The intervention was delivered most successfully by the case manager with a pre-existing interest and skills in dementia care. While the gap between the existing skills of the case managers and those required for the role could have been mediated through training and supervision, this was not feasible within the resources available. Existing line management arrangements continued for all case managers but seemed inadequate to support them in their new role. The time allocated for mentoring and the geographical dispersion of the case managers, meant that the number of face-to-face meetings with the mentor was limited and support was sometimes provided by telephone or email; closer supervision of the case managers may have facilitated a more consistent approach to case management:

*“If it was going forward the induction would be more protracted and would include joint visits, one where perhaps based on their own levels of knowledge or need maybe the supervisor either contributes to that initial assessment or supports them more proactively in that assessment, and then some time for reflection and feedback and recording afterwards; it would be much tighter than we were able to do.”* (Mentor)

A key training need that emerged at a relatively late stage of the study related to the ability of the case managers to identify and act on needs. Discussions between the researchers indicated that the unmet needs identified in the formal baseline assessments conducted by the research team were not always identified in the subsequent assessment by case managers. Furthermore, even where needs were identified and recorded, case managers did not always take appropriate actions. These discrepancies were observed for all of the case managers.

Several factors seemed to contribute to the difficulties case managers experienced in identifying unmet needs including the limited contacts between the case managers and patients and carers. The mentor had a background in Admiral Nursing and stressed that a series of visits was often required in order to understand a family’s needs:

*“when I’ve worked with case management it will have sometimes taken two or three visits, unless somebody has been in crisis, to identify what the true needs are of the relative and the person with dementia because that first meeting is sort of meet and greet and explaining why you’re there and then through that engagement and then building that relationship they’ll trust you enough to either tell you or least enable you to identify what the needs are and I don’t feel that that ability to build that relationship was there because of the time.”* (Mentor)

Patients and carers often explicitly stated during the qualitative interviews that they did not have any specific needs or problems:

*“We haven’t, luckily we haven’t had any major problems; it’s just day to day things.”* (Carer, B02)

Some carers, however, commented on the difficulties of recognizing their own needs; other participants acknowledged their reluctance to ask for help. While patients and carers often described the case manager as providing a ‘safety net’ this was only really effective with regular contact and monitoring:

*“You need somebody to be able to look at the bigger picture, who knows where you’re going, who’s seen it before and deem and assess your situation to be stable and tenable or not and either talk to you about it, get you the right support or what have you, but you can’t be the judge of your own situation. I mean obviously you know it’s bad, but sometimes you just don’t know what to do.”* (Carer, A04)

*“I wouldn’t personally ask. I’m happy to accept it all, if somebody points me in the right direction, I just won’t initially ask. I mean I wouldn’t say to you, ‘I’m struggling with this’.”* (Patient, C02)

### Reflexive monitoring: appraising and embedding case management

For many stakeholders, the intuitive appeal of case management overrode the lack of information on outcomes and the difficulties experienced with implementation. Thus they remained convinced that it was the ‘right’ intervention, but had failed due to various reasons, rather than fundamentally questioning whether the adapted intervention was appropriate for English health and care systems.

#### Assessing the impacts of case management

Whilst patients and carers gave positive feedback on the interpersonal skills of case managers, for example, describing them as ‘nice’, ‘pleasant’, ‘bubbly’, ‘lovely’, ‘easy to talk to’, ‘a friendly face’, ‘comforting’ and ‘supportive’, they found it difficult to evaluate the extent to which the anticipated benefits of case management had been realised in practice. Patients and carers were generally satisfied with their experiences of case management and several participants wished the service to remain in place (to benefit both themselves and others). The intervention created feelings of security or comfort for some patients and carers, and some practical benefits were reported:

*“What was very useful was when I told her [case manager] that trying to get appointments is really difficult; she’s actually used a pop-up system now in the surgery to get the earliest appointment without me having to say ‘is it possible, can you bring the appointment a bit forward?’ .”* (Carer, A06)

While a potential benefit of being based in primary care was the ability of the case manager to liaise with colleagues in the event of concerns about individual patients and carers, there was little evidence of this happening in practice. GPs were often unaware of which patients and carers had received the service:

*“To tell you truthfully, I have no idea if you had picked the two, the people up that I referred and I have no idea what has been done with them on behalf of the project and I have no idea if it’s made a difference.”* (GP, C03)

Even in GP practices where existing practice nurses took on the role of case manager, it was unclear exactly how familiar their colleagues were with their activities. Health and social care professionals outside primary care similarly found it difficult to assess the impact of case management beyond having received a small number of referrals or requests for information from the case manager.

#### The right intervention but at the wrong time

Several stakeholders felt that case management was the ‘right’ intervention, but for many clients had been delivered at the ‘wrong’ time in the illness trajectory. People with dementia and their carers who had only recently been diagnosed often felt that they didn’t need any support at the moment, but envisaged a time in the future where they would value case management. In contrast, patients and carers at a later stage in the illness trajectory sometimes reported that the intervention had come too late:

*“At the moment you see with my wife things are in early stage, aren’t they? So you know we might be very, very glad of [case manager] in months, years, a couple of years to come you know, I hope she’s still about to help us.”* (Carer, B03)

*“The things that [case manager] had to offer were perhaps something that I would have found very useful at the beginning of my mum’s Alzheimer’s and not so much [now] because I’ve learnt by trial and error on how to deal with it.”* (Carer, C01)

Professionals generally thought that case management ideally should be introduced either at diagnosis or at the point at which professionals involved in diagnosis withdrew:

*“Probably it should happen at diagnosis so that the patient and the carers are aware that the service is there without being awfully intrusive… So I would have thought from the word go … and then we go in say once every three months… or even once every six months depending on what they’re like.”* (GP, C04)

#### Embedding case management in practice

In terms of sustainability of the intervention in practice, the advantage of skilling-up a member of the practice team was apparent at the end of the study. In the two practices where a social worker had been seconded to the team, there were no plans to continue case management. In contrast, both practices with practice nurse-led case management intended to continue it in some form, although no clear arrangements or resources had been identified in one practice. In the other practice, the practice nurse was to continue one session a week on dementia-related activities such as maintenance of the practice dementia register and annual dementia patient reviews although she was hoping to continue with some aspects of case management:

*“I think it’s kind of just flagged me up as being a member of the team who does have the special interest, does have some skills that I can bring to the practice with, concerning dementia care and now that’s kind of got the ball rolling with me doing the [practice dementia register] and reviewing patients that way.”* (Case manager 1)

A common suggestion for improving integration of case management was for case managers to provide regular feedback to their colleagues in primary care and local community services. It was thought this would help clarify role boundaries and facilitate joint case work. Several existing meetings were identified in which such communication could potentially take place, including mental health team meetings, community nurse meetings or multi-disciplinary practice team meetings:

*“Every 3 months we have a meeting with [name] who is our consultant psychiatrist and I think that could be a useful tag on to that. It could be, if that was inconvenient, it could be a tag on to our regular meetings with the district nurses.”* (GP, C01)

## Discussion

This paper uses the lens of NPT to explore why a successful case management intervention from the US proved difficult to implement in primary care in England. We identified multiple barriers to implementation which reflected the full range of constructs within NPT and highlight the importance of both personal and system level factors. A fundamental problem concerned a lack of clarity over the nature and scope of the intervention. Whilst case management was intuitively appealing and therefore had theoretical *coherence;* all stakeholders in this study were unclear as to what the intervention involved in practice. The US study from which our intervention was adapted specified minimum levels of intervention, both in terms of the number of contacts and content to be delivered (e.g. education on communication skills); in our model giving case managers more autonomy over these aspects of intervention delivery, together with the time constraints of two of the case managers, may have contributed to the lower levels of case management implementation we observed. Although a detailed job description was produced [13] which was available to local services, this did not allay uncertainty over boundary issues; as in previous studies existing professionals (e.g. community mental health nurses and dementia advisors) perceived themselves as providing a similar service [16]. It has been suggested that future studies should provide a detailed description of the scope and nature of the case management delivered to facilitate interpretation of the findings [24]; such information could also help stakeholders understand how case management fits with existing services.

There was a mismatch between espoused investment in case management (*cognitive participation*) and practical resources and support both for individual case managers and participating practices. The ‘fit’ of case management with the culture of primary care was questioned, and it was clear that case managers needed robust time management skills in order to manage this tension. We confirmed that both ‘expert knowledge’ of dementia, ‘conviction’ and individual commitment contributed to successful delivery of case management [16,25].

Implementation in practice (*collective action*) was hindered by reports of limited time, insufficiently robust supervision arrangements and the constraints associated with a feasibility study. Variability in time allocated to case management has previously been highlighted [8] and it has been suggested that more intense programmes are associated with better outcomes [7]; in our study, a low intensity service was provided by all case managers, regardless of the time available [14]. A key barrier which has not previously been identified concerned the ability of case managers to identify, and act on, emerging patient and carer needs; we identified examples of missed and unmet needs for all three case managers [14]. One case manager explicitly attributed this to the timing of the intervention; a study of case management for people with early symptoms of dementia and their carers similarly found that case managers did not feel the intervention was needed at this point [15]. Despite extensive and recent reviews on the effectiveness of case management, there remains a lack of consensus over the appropriate timing of this intervention along the dementia pathway [1,16,26]; some authors have suggested that case management should be introduced from the onset of dementia, even before diagnosis, but evidence to support this is limited, with some studies suggesting that more cognitively impaired patients benefit more from case management [6]. Since a key characteristic of case management is to prevent problems and initiate early intervention [24] additional training may be required to ensure that professionals achieve this in practice. This may particularly be the case for professionals who are used to working in contexts (e.g. social care, care homes) with high eligibility criteria for support.

While integration of case management within the local dementia care network has been identified as a key factor for success [7,16,24], this was not achieved in the present study. Although a range of integration mechanisms were identified by health care professionals, these only emerged towards the end of the study. More formal monitoring of integration and implementation may have facilitated early recognition of problems and allowed for modification [25]. Support and supervision arrangements for case managers may also need more specification. In the US study from which our intervention was adapted case managers met weekly with a geriatrician, geriatric psychiatrist and psychologist to review cases. In the present study we provided a degree of ‘distance mentoring’ but anticipated that the case managers would act as autonomous practitioners. A previous study of case management for older people suggested that a ‘hands off’ management style may have encouraged inertia and a lack of interest [25]. With more robust supervision arrangements, either via a practice dementia champion or the community mental health team, it is possible that the case managers could have achieved more within the time and resources available. This study confirms that effective implementation of case management is dependent on not only having the right people, with the necessary skills and support, but the right context, which enables successful integration into the broader existing care system.

In three of the four practices, there was little evidence that case management had been embedded in practice (*reflexive monitoring*). In one practice, however, there were plans to continue a modified version of the intervention. This relative success seemed to primarily reflect the enthusiasm and commitment of the practice nurse who took on the case manager role.

### Limitations of the study

The limitations of the study relate to the lack of attention paid to implementation strategies and the time limited nature of a pilot study; the latter meant we were unable to address the question of the most appropriate time to implement case management in the dementia pathway. The process of adapting the US intervention for primary care in England, focused primarily on developing appropriate written materials, a job description, person specification and educational needs assessment [13]. While these documents specified a range of tasks, monitoring of progress focused primarily on recruitment, with little attention to other aspects of intervention fidelity. The qualitative interviews with health care professionals tended to take place towards the end of the study in order to obtain a retrospective view of case management; this meant that some of the problems with implementation emerged only towards the end of the study. Similarly, a review of case manager notes was conducted at the end of the study as a result of the perceived mismatch between needs identified through the formal assessments conducted by the research team and those identified by case managers. The process evaluation therefore had limited scope to inform implementation. The study was further affected by significant changes in the organisation of primary care services in England during the study period.

The interviews with case managers, the mentor and team members were, in general, based on first-hand experience of case management (or an aspect of case management of which the participant had specific knowledge). In contrast, the lack of visibility of case management to colleagues both within and outside the primary health care team, meant that other stakeholders had limited knowledge of the intervention. Patients and carers were able to describe their experiences of assessment and any subsequent contacts with the case manager. However, many of their comments on the value of case management related to hypothetical or potential benefits of this approach rather than personal experience. Health and social care professionals, even those within participating practices, also reported a lack of contact with case managers and limited knowledge of their work. The data therefore are skewed towards abstract views of the concept of case management, rather than practical experiences of this approach.

### Implications for practice and future research

Our feasibility study found that despite the initial enthusiasm of participating GP practices and adequate funding for intervention delivery, little case management was implemented. The reasons for this were similar to those found in previous studies [7,15]. To embed case management into real world practice requires considerable attention to both developing and supporting the case manager and also the work needed to embed this new role into existing care systems. In addition, a key requirement for case manager is to exert a low threshold for addressing client need [16]. Protected time is also a crucial factor [27] as well as supervision to facilitate transition to the role of case manager. Although our participants felt primary care to be the appropriate location for the case manager, it may be that in England, where primary care has recently undergone considerable organisational upheaval with GPs becoming service commissioners as well as health care providers, that well-established community mental health teams in secondary care may have afforded a more appropriate environment (although these too are undergoing organisational change [28]). Our study further highlights the importance of adequately resourcing facilitation of implementation alongside the research [29].

The most common needs of people with dementia and their families are consistently found to be social and psychological [30,31], yet our case managers had difficulties in identifying and addressing these needs. The ‘day to day things’ described by patients and carers tended either not to be raised or not to be seen as a legitimate focus of attention by case managers, suggesting that additional training is needed to address this. In the present study individual case managers undertook a needs assessment prior to their one day induction course [13]; however this relied on them being able to identify and articulate their training needs. More comprehensive and longer training may be required to equip case managers with the necessary skills [32].

## Conclusion

In conclusion, while case management intuitively appealed to all stakeholders as a proactive approach to addressing unmet needs, we encountered multiple barriers to the implementation of case management for dementia in primary care in England. There was limited scope in the present study to address many of the issues identified by the process evaluation (e.g. supervision arrangements, time available, integration mechanisms, most appropriate timing for intervention). The role, timing and site for case management for people with dementia in England remains unclear and further research is required. By establishing the conditions required for the successful implementation of case management, we hope the findings of the present study will facilitate future studies.

## Endnotes

^a^Admiral Nurses are mental health nurses specialising in dementia who support families throughout the dementia journey and facilitate co-ordinated care between different parts of the health and social care system. The availability of Admiral Nurses varies throughout the UK. For more information see www.dementiauk.org.

^b^Practice nurses are qualified and registered nurses who work in GP surgeries as part of the primary healthcare team.
